# Prediction of Violence, Suicide Behaviors and Suicide Ideation in a Sample of Institutionalized Offenders With Schizophrenia and Other Psychosis

**DOI:** 10.3389/fpsyg.2018.01385

**Published:** 2018-08-07

**Authors:** Miriam Sánchez SanSegundo, Rosario Ferrer-Cascales, Jesús H. Bellido, Mar P. Bravo, Javier Oltra-Cucarella, Harry G. Kennedy

**Affiliations:** ^1^Department of Health Psychology, University of Alicante, Alicante, Spain; ^2^Department of Psychology, Alicante Forensic Psychiatric Hospital, Alicante, Spain; ^3^Department of Psychiatry, Institute of Legal Medicine, Alicante, Spain; ^4^Department of Psychiatry, Trinity College, University of Dublin, Dundrum, Ireland; ^5^Central Mental Hospital, Dublin, Ireland

**Keywords:** suicide, violence, risk assessment, schizophrenia, HCR-20

## Abstract

This study examined the predictive validity of the Spanish version of the Suicide Risk Assessment Manual (S-RAMM) and the *Historical-Clinical-Risk Management-20* (HCR-20) in a sample of violent offenders with schizophrenia and other psychosis, who had committed violent crimes and had been sentenced to compulsory psychiatric treatment by the criminal justice system. Patients were prospectively monitored within the institution for 18 months. During the follow-up period, 25% of offenders were involved in any suicidal behavior including acts of self-harm, suicidal ideation and suicide attempts and 34% were physically or verbally violent. The S-RAMM and HCR-20 risk assessment tools were strongly correlated and were able to predict suicidal behavior and violence with a moderate-large effect size (AUCs = 0.81–0.85; AUCs = 0.78–0.80 respectively). Patients scoring above the mean on the S-RAMM (>20-point cut-off) had a five times increased risk of suicide related events (OR = 5.05, 95% CI = 2.6–9.7) and sevenfold risk of violence in the HCR-20 (>21-point cut-off) (OR = 7.13, 95% CI = 2.0–21.2) than those scoring below the mean. Offenders at high risk for suicide and violence had significantly more suicide attempts (*p* < 0.001) and more prior sentences for violent crimes (*p* < 0.001). These results support the use of the S-RAMM and HCR-20 for clinical practice by providing evidence of the utility of these measures for predicting risk for suicidal and violent behavior in mentally disordered offenders.

## Introduction

Suicide is one of the leading causes of premature death worldwide among patients with schizophrenia (Kasckow et al., 2014). Between 4 and 5% of patients with schizophrenia and other psychoses complete suicide (Hor and Taylor, 2010), between 40 and 79% report suicidal ideation, and between 20 and 40% of these individuals make suicide attempts during the course of illness (Skodlar et al., 2008; Gill et al., 2015). An increased risk of suicide in schizophrenia has been commonly associated with factors such as mood disorder, previous suicide attempts, or drug misuse, some of them being shared with the general population (Popovic et al., 2014) and some being specifically related to this disorder (Siris, 2001; Hawton et al., 2005). In a systematic review of risk factors for schizophrenia and suicide, Hawton et al. (2005) identified seven risk factors for suicidal behaviors including the presence of depressive disorders, previous suicide attempts, drug misuse, agitation, fear, poor adherence to treatment and recent loss. Suicide has also been associated with the presence of active delusions and hallucinations, particularly among violent offenders with schizophrenia and other psychosis (Hor and Taylor, 2010). However the risk of suicide among these patients remains relatively constant throughout the life-span (Bhatia et al., 2006) and is particularly high during hospitalization and immediately following discharge (Qin and Nordentoft, 2005; Meehan et al., 2006).

Identifying risk factors for suicide and self-harm has been established as the best strategy for predicting and preventing suicide and other adverse (Pompili et al., 2007). However the prediction of risk for suicide has been considered an imprecise and complex process leading to many false positive results (Ishii et al., 2014).

In recent years there has been a growing interest in improving the accuracy of violence risk assessment by using the Structured Professional Judgment (SPJ) approach, which provides more accurate predictions than unstructured clinical assessments (Singh et al., 2014). Such schemes provide guidelines for assessing risk based on empirical risk factors that are amenable to clinical interventions and are coded in a flexible way in order to enhance a decision (Douglas and Skeem, 2005). However, while there are well validated tools for assessing risk of violence in mentally disordered populations (Webster et al., 1997), little effort has been paid to validate tools for assessing the risk for suicide (Ijaz et al., 2009). In addition, the few existing research studies on suicide in individuals with schizophrenia and other psychoses are restricted to patients in the community or in community hospitals, but much less is known about violent offenders admitted to secure psychiatric hospitals and correctional settings (Horon et al., 2013; Shibre et al., 2014). Some recent studies suggest that exposure to the criminal justice system contributes to elevating the suicide risk especially among people sentenced to psychiatric treatment and among those experiencing multiple contacts or with a history of charges for violent offenses (Webb et al., 2011). Suicide risk among these populations has been found to be between 11 and 14 times greater than in the general population (McKee, 1998; Lekka et al., 2006) and is more prominent among individuals experiencing feelings of guilt (Dooley, 1990), isolated or confined in a single cell (Lekka et al., 2006), and sentenced to long-term detention after committing violent crimes (DuRand et al., 1995). Also, while most studies to date have examined the rates of suicide and violence separately, it has been reported that both behaviors often co-occur in the general population as well as in mentally disordered offenders (Witt et al., 2014). Violence in schizophrenia and other psychosis has been significantly associated with hostile behavior, poor impulse control, lack of insight, drug misuse, past history of criminality and non-adherence with medication (Witt et al., 2013). Some common risk factors such as levels of symptomatology, hostility, impulsivity and lack of compliance with medication may be related to suicide and violent behaviors and exacerbate these tendencies whose course may run in parallel (Witt et al., 2014).

The aim of the present study was to examine the predictive validity of the Spanish Version of the Suicide Risk Assessment and Management Manual (S-RAMM; Bouch and Marshall, 2003) and the HCR-20 V2 (Webster et al., 1997) for predicting violence toward self and others in a sample criminal offenders with Schizophrenia and other Psychosis. To our knowledge, the S-RAMM is the first SPJ tool developed for identifying risk factors associated with suicide and self-harm and for planning risk management. The S-RAMM has been reported to have a good inter-rater reliability, internal consistency and discriminative ability for distinguishing between levels of security within secure psychiatric hospitals (Ijaz et al., 2009). The S-RAMM has also been found to be a valid and feasible measure for predicting self-harm and suicidal behaviors with areas under the receiver operating curve (AUC) from 0.79 to 0.99 for periods of follow-up of 6 months (Fagan et al., 2009).

We hypothesized that the Spanish version of the S-RAMM would perform similarly to the original English version for the prediction of suicidal behaviors in criminal offenders. Also, because of evidence showing that suicide and violence often co-occur in this population (Hunt et al., 2006; Suokas et al., 2010; Witt et al., 2014), we investigated the association between suicidality and violence by using the *The Historical-Clinical-Risk Management- 20* (Webster et al., 1997), a well validated tool for the SPJ of risk of violence in mentally disordered populations. To test these hypotheses, we report a prospective longitudinal study of 18 months follow-up in a criminal sample of patients with schizophrenia and other psychoses who had committed violent crimes and had been sentenced to compulsory psychiatric treatment.

## Materials and Methods

### Participants

The study was conducted at the Forensic Psychiatric Hospital of Alicante (Spain) which provides medium and maximum-security for all violent offenders admitted from the Spanish courts or transferred from prisons because of a mental disorder. The institution has 375 beds for violent offenders with major mental disorders, personality disorders and mental disability. At the time of the study there were 250 patients at the institution out of 400 mentally disordered offenders detained across Spain. Around 30% of patients (*N* = 75) were admitted under psychiatric orders after committing murder or homicide (for a more detailed description of Spanish procedures see Sánchez-SanSegundo et al., 2014).

The initial sample included 82 mentally disordered violent offenders who were part of a large study of neuropsychology and recidivism carried out at the institution. Participants were included in the study if they: (i) had a primary clinical diagnosis of severe mental illness including schizophrenia, schizoaffective disorder, delusional disorder and other psychosis according to the Diagnostic and Statistical Manual of Mental Disorders (DSM-IV-TR, 4th American Psychiatric Association, 1994); (ii) had committed at least one criminal offense leading to compulsory psychiatric admission, (iii) had been found not criminally responsible by reason of insanity by the Spanish criminal justice system. Participants were excluded if they (i) had severe symptoms of psychopathology as defined by a score ≥ 3 in the *Positive and Negative Syndrome Scale* (PANSS) at the moment of assessment that would affect their ability to answer the questions during the interview (Peralta and Cuesta, 1994), (ii) had been declared incapacitated or legally incompetent by the Spanish civil law, or (iii) their primary language was not Spanish. Of the 82 initial patients eligible to participate in the study, 5 (6.1%) were excluded due to active psychopathology, 19 (22.8%) refused to take part, 4 (4.8%) were transferred to prison or discharged to the community during the follow-up, 3 (5.8%) could not be scored due to missing items on the risk assessment tools. Finally 51 (62.2%) patients formally consented to take part of the study. The majority of the sample (72%; *n* = 38) was charged with murder or homicide and were subsequently sentenced to compulsory psychiatric treatment. The patients were 45.6 years old on average (*SD* = 8.3). Thirty-three patients (63.6%) met criteria for schizophrenia, 7 (13.7%) for delusional disorder, 7 (13.7%) for schizoaffective disorder, and 4 (7.6%) for other psychotic disorders. In addition, 12 patients (22.5%) met criteria for a comorbid diagnosis of personality disorder and 32 (60.8%) met criteria for substance dependence or abuse. Using the *Global Assessment of Functioning* (GAF; Jones et al., 1995) the majority of patients were moderately ill (*n* = 35, 68.6%) at time of the study with a mean score of 52.14 (*SD* = 13.92). Most participants had a long previous history of psychiatric treatment with at least two or more prior contacts with Mental Health Services (*n* = 46, 91.9%) and a previous history of suicide attempts (*n* = 29, 57.1%). The most common offenses leading to compulsory treatment were murder or attempted murder (36.6%) followed by homicide or attempted homicide (34.7%) and other severe violent offenses, including assault (11.7%), sexual offense (5.7%), and violent threats of death (9.6%). The average length of stay at the institution at the start of the study was 143 months (*SD* = 81.12, range 6–360 months). The majority of patients had committed their offenses against family members or known victims (71.2%), and 13.4% had been physically or sexually abused as children.

### Ethical Approval

The present study was approved by the Ethics Committee of the Alicante Forensic Psychiatric Hospital (HPPA-2885/431-2014) and it was conducted according to the Ethical Principles for Medical Research Involving Human Subjects (Declaration of Helsinki, 1964). All participants were mentally capable and legally competent to give written informed consent according to the Spanish Civil Law Procedure (art.293). Patients were informed that their answer would have no negative consequences and would not affect their privileges, restrictions or treatment.

### Measures

#### S-RAMM. Suicide Risk Assessment Manual

The S-RAMM (Bouch and Marshall, 2003) is a SPJ tool designed for assessment of risk of suicide. The instrument provides a structured approach to determining the level of suicide risk and the issues that need to be addressed for planning risk management. The S-RAMM is closely modeled on the HCR-20 and contains 22 risk factor items grouped into three scales: 9 Background Risk Factors (B), 8 Current Risk Factors (C) and 5 Future Risk Factors (F). Each item is scored on a three-point scale indicating the presence (2), possible presence (1) or absence of each risk factor (0). Higher scores indicate higher risk of suicide. The English version has adequate inter-rater reliability and internal consistency values, with Cronbach’s alpha above 0.8 for the total score (Ijaz et al., 2009). The S-RAMM has also been found to be an excellent tool for predicting self-harm within forensic psychiatric institutions [Area Under the Curve (AUC) = 0.89, IC 95% 0.79–0.99] (Fagan et al., 2009). Psychometric properties of the Spanish version of the S-RAMM have also been shown to have adequate inter-rater reliability and adequate internal consistency for all subscales and for the total score, with Cronbach’s alpha values above 0.89.

#### HCR-20. The Historical-Clinical-Risk Management-20

The HCR-20 V2 (Webster et al., 1997) is a SPJ tool designed for the assessment of risk of violence. The instrument contains 20 risk factors grouped into three scales: Historical (H), Clinical (C) and Risk Management (R). Factors are scored on a three-point scale (Dawes, 1979) indicating the presence (2), possible presence (1) or absence of each risk factor (0). Higher scores with an increasing number of risk factors indicate higher risk of violent acts. A final clinical risk judgment is provided as low, moderate or high risk, indicating the specific interventions aimed to manage violence risk. The psychometric properties of the instrument have been examined in numerous studies reporting rates of moderate to excellent predictive validity [see Douglas and Reeves (2010) for a review]. Psychometric properties of the Spanish version of the HCR-20 have also been shown to have adequate interrater reliability and predictive validity for violent offenses (AUCs = 0.69–0.77) in chronic psychiatric populations (Arbach-Lucioni et al., 2011).

### Outcome Measures

Adverse events of suicide and violence were prospectively monitored within the institution over an 18 month follow-up period. Violence incidents within the institution were prospectively monitored by the staff observation using the Spanish version of the Modified Overt Aggression Scale (MOAS; Arbach-Lucioni et al., 2011), a non-intrusive, observational scale designed to assess the frequency and severity of aggressive behavior. Violence was defined according to HCR-20 manual as “actual, attempted or threatened physical harm deliberately to others.” This definition allows inclusion of harmful or injurious acts to others as well as property damage with the goal to frighten or threaten another person, verbal threats, insults, intimidation, and other behaviors perceived as malevolent and intended to induce fear or to harm others. For the purpose of the present study, verbal threats and physical violence directed toward others were both considered as violence incidents. From these two, a composite outcome measure of “any violence” was derived. Suicidal behaviors were classified into two broad categories including acts of self-harm defined in the S-RAMM Manual (item B1) as “attempted suicide or self-injury which includes a range of behaviors between low and high suicidal lethality” and “suicidal ideation, communication or intent” defined as any self-reported thoughts of committing suicide (S-RAMM item C1). A combined measure of suicidal behaviors was then derived from the sum of both items.

### Procedure

Demographic, clinical and criminal variables were collected from each patient’s hospital files. Participants were interviewed individually prior to the beginning of the follow-up period. Semi-structured interviews took from 2 to 3 h and included the administration of the S-RAMM and HCR-20 risk assessment measures.

Outcomes for suicidal and violent behaviors were then monitored and registered during 18 months of follow-up within the institution (from May 2014 to August 2015). Incidents were collected by staff observations as part of the clinical routine and from the incident reporting systems. An independent forensic psychologist (JH) who was blind to the scores on the predictions, recorded acts of suicidal and violent behavior.

### Statistical Analysis

Correlations between the S-RAMM and the HCR-20 were examined with Spearman’s rank correlation coefficient, a non-parametric measure. Participants were divided into two groups using the score above the mean on the S-RAMM and the HCR-20. Bootstrapping ROC curve using the web-bootstrap free software (Skalská and Freylich, 2006) with *k* = 3000 simulated random samples were used to examine the predictive accuracy of the S-RAMM and HCR-20. ROC analysis has been shown to be a valid method in research for the prediction of suicidal behavior and violence using such scores because it is much less sensitive to base rate than other procedures (Mossman, 1994). It reflects the probably that any individual will be correctly classified. AUC values range from 0 to 1, where an AUC of 0.50 represents chance-level prediction and an AUC of 1.00 represents perfect predictions. In general, AUC values of 0.70 and above are considered indicative of moderate to large effect size while values above 0.75 are interpreted as large (Douglas et al., 2005). Odds ratios were calculated to detect differences in suicidal and violent behaviors between patients scoring above the mean and patients scoring below the mean separately for the S-RAMM and HCR-20.

## Results

### Outcome Events for Suicide Events

Out of 51 patients in the cohort, 13 (25.5%) were involved in any incident of self-harm, ideation, communication or attempt of suicide during 18-months follow-up. A total of 6 (11.7%) committed self-harm, including one completed suicide and one suspected death by suicide, while 11 (21.5%) had incidents of suicidal ideation, intent or communication. The S-RAMM final structured risk judgements classified 16 (31.3%) of the patients as high risk, 19 (37.2%) as moderate risk and 16 (31.4%) as low risk.

### Outcome Events for Violence Behaviors

For violence, 18 (35.29%) patients were involved in any violent incident during the follow-up period at the institution. Thirteen individuals (25.5%) showed acts of aggression against other patients or members of the staff, while 17 (33.3%) showed verbal aggression including violent threats of death. The HCR-20 final structured risk judgements classified 20 (39.2%), 15 (29.4%) and 17 (33.3%) patients as high, moderate and low risk respectively. The proportion of patients who were violent across the follow-up differed significantly across structured final risk categories (*p* < 0.001).

#### Differences Between Offenders With and Without Aggressive and Suicide Behaviors

The percentage of patients who behaved violently and showed any incident of self-harm, ideation or communication of suicide across the follow-up was 84.6% (*n* = 11). Compared with non-suicidal patients, patients who were involved in any suicidal event showed higher total scores on the S-RAMM (*t* = -3.6; *df* = 49; *p* < 0.001; *M* = 25.8 [*SD* = 5.09] vs. *M* = 18.0 [SD = 7.06]) and higher total score on the HCR-20 for violence incidents (*t* = 2.5; *df* = 49; *p* < 0.01; *M* = 25.1 [*SD* = 6.79] vs. *M* = 18.9 [*SD* = 8.47]). Patients scoring above the mean on the S-RAMM (>20-point cut-off) and HCR-20 (>21-point cut-off) had a five times increased risk of suicidal behavior (OR = 5.05, 95% CI = 2.64–9.70) and sevenfold risk of reoffending (OR = 7.13, 95% CI = 2.0–21.2) than those scoring below the mean. Furthermore, patients scoring above the mean on the S-RAMM and HCR-20 total score had significantly more suicide attempts (*M* = 4.3 vs. *M* = 1.0, *z* = -3.7, *p* < 0.001) after the baseline assessment. They also had more prior sentences for violent crimes (*M* = 6.2 vs. 3.5, *z* = -4.23, *p* < 0.001). No significant differences between violent and suicidal patients and non-violent and non-suicidal patients were found with respect to educational level (*p* ≥ 0.69), marital status (*p* ≥ 0.69), and history of drug misuse (*p* ≥ 0.08).

### Predictive Validity of the S-RAMM for Suicide Events

**Table 1** shows the results of the predictive validity of the S-RAMM for suicidal behaviors.

**Table 1:** Bootstrapping ROC Curves for suicide behaviors by using the S-RAMM risk assessment tool.

*k* = 3000	Any suicide behavior				Self-harm				Suicide Ideation or communication			
	AUC	*SE*	IC 95%	*r*	AUC	*SE*	IC 95%	*r*	AUC	*SE*	IC 95%	*r*
S-RAMM total	0.84	0.05	0.74–0.93	0.54^∗∗^	0.81	0.07	0.65–0.93	0.35^∗∗^	0.84	0.05	0.74–0.93	0.52^∗∗^
B SRAMM	0.73	0.07	0.57–0.89	0.36^∗∗^	0.61	0.06	0.51–0.79	0.31^∗^	0.73	0.06	0.57–0.89	0.34^∗^
C SRAMM	0.76	0.06	0.61–0.89	0.41^∗∗^	0.77	0.07	0.60–0.88	0.30^∗^	0.78	0.06	0.65–0.89	0.42^∗∗^
F SRAMM	0.75	0.06	0.62–0.87	0.40^∗∗^	0.74	0.07	0.59–0.86	0.28^∗^	0.72	0.07	0.60–0.86	0.35^∗^

*AUC, area under the receiver operating characteristic curve; oxS-RAMM, Suicide, Risk, Assessment Manual; CI, confidence interval; ns, not significant.*

**FIGURE 1:**
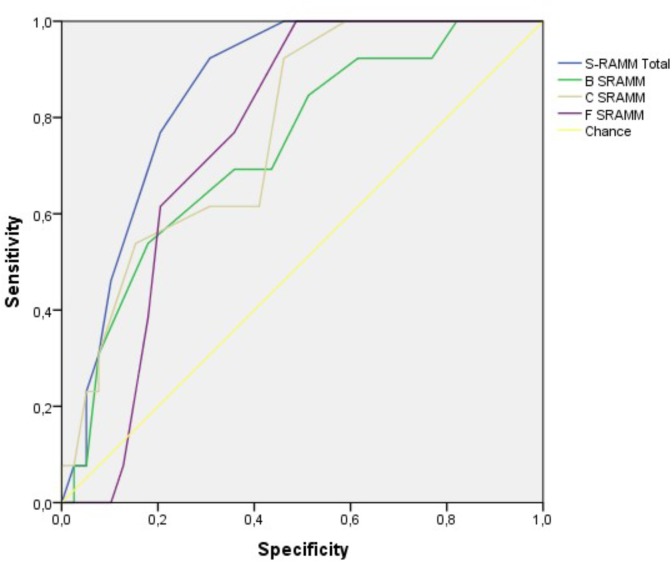
ROC curve analysis for suicide events.

For all forms of suicidal behavior, the S-RAMM scores showed a moderate-large AUC ranging from 0.84 (for any suicidal behavior and suicide ideation), to 0.80 (for self-harm) and correlations for suicide ranging between 0.35 and 0.54. All the S-RAMM subscales produced a similar pattern of correlations and predictive values with little difference between the subscales (**Figure 1**). Using as cuttof the score above the mean (<20) on the instrument, the sensitivity for any suicide behavior was 70% and the specificity was 87%. The positive predictive value (PPV) was 64% and the negative predictive value (NPV) was 89%. For the criteria suicide attempts value for sensitivity was 67%, specificity 78%, PPV 28%, NPV 94%.

### Predictive Validity of the HCR-20 for Violent Behaviors

The predictive ability of the HCR-20 total score for inpatient aggression was large, with AUC values of 0.78 for threatening behaviors and 0.80 for physical acts of violence (*r* = 0.47–0.41). For the combined measure of any violence, the HCR-20 total score was a significant predictor of violence (AUC = 0.85; *r* = 0.49) (**Tables 1, 2**).

**Table 2:** Bootstrapping ROC Curves for suicide behaviors by using the HCR-20 risk assessment tool.

*k* = 3000	Any violence				Physical violence				Threatening behavior			
	AUC	*SE*	IC 95%	*r*	AUC	*SE*	IC 95%	*r*	AUC	*SE*	IC 95%	*r*
HCR 20 Total	0.80	0.06	0.66–0.92	0.49^∗∗^	0.79	0.06	0.67–0.89	0.41^∗∗^	0.78	0.06	0.65–0.90	0.47^∗∗^
H HCR	0.78	0.06	0.65–0.90	0.46^∗∗^	0.78	0.06	0.64–0.89	0.37^∗∗^	0.77	0.07	0.64–0.89	0.45^∗∗^
C HCR	0.75	0.06	0.60–0.87	0.43^∗∗^	0.76	0.06	0.63–0.88	0.31^∗^	0.73	0.06	0.61–0.87	0.40^∗∗^
R HCR	0.71	0.07	0.54–0.81	0.35^∗^	0.71	0.07	0.57–0.87	0.26^∗^	0.70	0.07	0.56–0.84	0.34^∗^

*AUC, area under the receiver operating characteristic curve; HCR-20, Historical, Clinical and Risk Management; CI, confidence interval; ns, not significant.*

**FIGURE 2:**
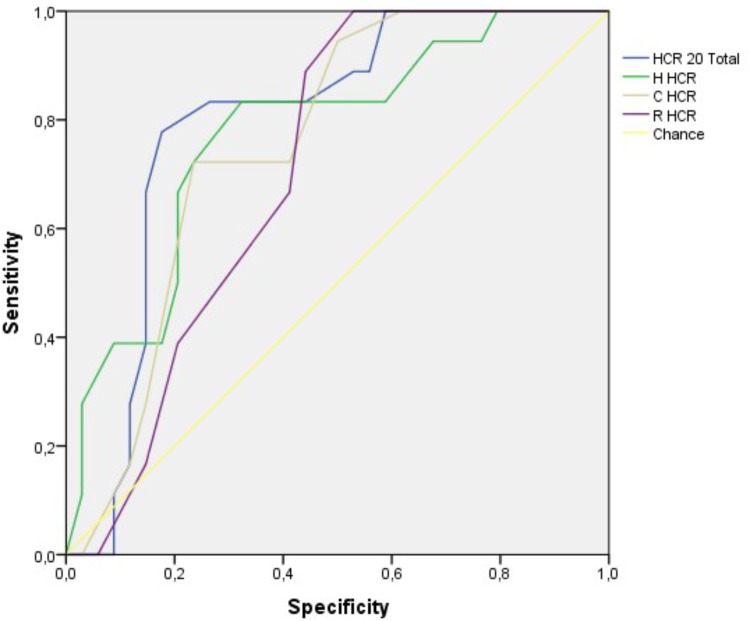
ROC curve analysis for violence events.

The AUC values for all subscales of the HCR-20 produced a moderate to large predictive validity with significant correlations for all subtypes of violent behaviors (**Figure 2**). Using as cuttof the score above the mean (<21) on the HCR-20, the sensitivity for any violent behavior was 73% and the specificity was 84%. The PPV was 60% and the NPV was 90%. For the criteria physical violence the value for sensitivity was 70%, specificity 75%, PPV 31%, NPV 90%.

## Discussion

We conducted a longitudinal prospective examination of the predictive validity of the Suicide Risk Assessment and Management Manual (S-RAMM) and the HCR-20 in a sample of mentally disordered violent offenders who had been found not criminally responsible by reason of insanity. As far as we know, the S-RAMM is the first SPJ tool validated in Spanish for identifying risk factors associated with suicide and self-harm with a view to planning risk management strategies.

We found that the S-RAMM was predictive of all forms of suicidal behavior over a period of 18 months. The S-RAMM total score was found to contribute most to the large effect size with AUC values ranging from 0.81 to 0.85. The (B)ackground, (C)linical and (F)uture subscales of the S-RAMM were independently related to suicide behaviors with little variation of AUC values between subscales. These results are similar to those reported by Fagan et al. (2009) in a prospective study examining the predictive ability of the S-RAMM over a short period of 6 months. They found that the S-RAMM was a good predictor of self-harm and suicidal behaviors in a sample of mentally disordered offenders with AUC values ranking from 0.79 to 0.99. The prevalence of suicidal behaviors observed in their study across 6-months of follow-up was 16%. The majority of patients reported incidents of suicidal ideation, intent or communication of suicide, with a percentage of 3.7% of patients showing intra-institutional acts of self-harm and completed suicide.

The base-rate of suicidal behaviors found in the present study was reasonably high. We reported that 25% of offenders in our cohort were involved in any suicidal behavior across the follow-up, including acts of self-harm, suicidal ideation and suicide attempts. However, the base-rate for suicide events dropped from 25 to 12% when the outcome criterion was restricted to self-harm and fatal suicide. We can speculate that the nature of the current study conducted within a controlled environment, characterized by restrictive access to methods of suicide might have inhibited the suicidal tendencies of patients at high risk who might be much more vulnerable to these behaviors in less restrictive settings (Buffington-Vollum et al., 2002; Endrass et al., 2008). A high prevalence of suicide and self-harm has been reported previously among offenders in psychiatric treatment in both forensic (Webb et al., 2011; Abidin et al., 2013) and correctional populations (Palmer and Connelly, 2005; Lekka et al., 2006; Fazel et al., 2008). Suicidal behavior in these populations has been considered as a continuum of increasing seriousness and lethality of behaviors, moving from thoughts, plans or wishes to self-injuries and fatal outcomes (Lekka et al., 2006). Some specific risk factors in forensic hospital and prison populations such as stresses of imprisonment, mental illness, and duration of custody may exacerbate this set of circumstances and contribute to increase risk for suicide (Palmer and Connelly, 2005). For example, Webb et al. (2011), in a national Danish case-control study of all suicide committed from 1981 to 2006 by people processed for any criminal charge found that a past history of psychiatric treatment was closely related with a more than 13-fold higher suicide risk in men and 25-fold increase in woman. They demonstrated that exposure to the criminal justice system contributed to elevating risk for suicide, especially among people sentenced to psychiatric treatment and among those with a history of violent offense charges (Webb et al., 2011). In addition, among people charged with violent offenses, intense feelings of regret and guilt may also play a key role in self-harming behaviors and suicide, particularly if offenses were committed against family members (Palmer and Connelly, 2005; Webb et al., 2011). We found that most patients in our cohort were charged with murder or homicide (71%) and the majority of violent offenses were directed toward family members or known victims (71%). Thus, a common conclusion of these findings is that both environmental and criminological risk factors may predispose mentally disordered violent offenders in custody to self-injurious behaviors and suicide.

The results of the present study also add support to the literature on violence that suggests that suicide and violence co-occur among patients with schizophrenia (Hunt et al., 2006; Suokas et al., 2010; Witt et al., 2014) and among forensic populations with these diagnoses (Webb et al., 2011). We examined the risk for violence in our cohort by examining the incident reporting system and staff observation. We found that 84.6% of patients who behaved violently showed at least one incident of self-harm, ideation or communication of suicide during the period of follow-up. Compared with non-suicidal patients, offenders who were involved in any suicidal behaviors showed higher scores above the mean in both the S-RAMM and HCR-20 risk assessment tools. As in previous studies, the HCR-20 was a good predictor of institutional violence in forensic and correctional settings (Douglas et al., 2003; Gray et al., 2003; McDermott et al., 2008).

The predictive models for suicide and violence showed good overall discrimination for predicting suicide and violent offending over 18 months within institution. However, both scales had better NPV than PPV values for identifying those patients at low risk of suicide and violence. These results are in line to those found by Fazel et al. (2017) who developed a web calculator (OxMIV) for risk of committing violent crime in a national cohort of individuals with schizophrenia and bipolar disorders. They found that the positive value of the OxMIV tool was 11% while the negative predictive value was more than 99% with a sensitivity of 62% and specificity of 94%. An important clinical implication of these findings is that while the risk assessment tools such as the S-RMM, HCR-20 and the OxMIV web calculator could be used to screen patients into low-risk and high-risk groups, it should be used with caution to predict suicide and violent crime in high-risk groups as only around six in 10 of those cases identified as high risk will commit an incident of violence, self-harm, ideation or communication of suicide. Thus, around 4 of 10 identified as high risk, will be misclassified by using the S-RAMM and HCR-20. Also, given that the use of risk assessment tools could overestimate the risk of violence and suicide in patients classified as at high-risk level, it should not be used to extend their detention of in the absence of other clinical evidence that support this conclusion (Fazel et al., 2017).

### Limitation and Future Directions

Despite of these findings, there are a number of potential limitations that need to be considered in future studies including the small sample size, the controlled environment of the study and the lack of validated scales for comparisons. Whether these measures can predict post-discharge incidents in forensic psychiatric patients in Spain will be needed to be examined in future studies. Also, it is not known how good the S-RAMM performs in contrast to other established suicide prediction tools in similar settings due to the lack of validated measures into Spanish. An additional limitation is that although adverse incidents of suicide and violence were carefully collected from staff observation and collateral reports from hospital record system, no published measures such as the Lethality of Suicide Attempt Rating Scale (LSARS-II; Berman et al., 2003) for determining lethality, number of prior suicide and method of die were used. Most important, while the mental health laws of most countries emphasize the importance of predicting suicidal behaviors among people in custody and among criminal offenders processed by the criminal justice system, the use of risk assessment for these purposes has been recently subject to criticism, particularly for rare and infrequent events (Large et al., 2011). It has been reported that for events with a low base rate (e.g., suicide, serious violence, and homicide), the predictive ability has not improved across 50 years of research (Franklin et al., 2016). It is widely recognized that the probability of predicting an event varies with the base rate of the group to which to the test is being applied (Janus and Meehl, 1997). Pavlou et al. (2015) suggest that when the number of events is low relative to the number of predictors in risk models, standard statistical procedures used in risk assessment research may produce over-fitted risk models, leading to a large number of “false positives.” The number of false-positive categorizations might be reduced by increasing the cut-off point of the scale (sensitivity), but inevitably it would produce an increased number of false-negatives (low specificity) (Mossman, 1994). In such cases, patients wrongly categorized into high or low-risk categories would result in dramatic consequences including: prolonged involuntary hospitalization, deprivation of liberty, more intensive supervision or lack of adequate treatment leading some ethicists to argue against allocating treatment resources according to risk assessment (Ryan et al., 2010; Large et al., 2011).

The use of innovative approaches such as “the ridge regression,” “lasso regression” and bootstrapping procedures have recently been suggested as a prominent alternative analyses for improving the accuracy of risk predictions in scenarios with low base rate of rare events (Pavlou et al., 2015). Also, new investigative models using non-linear dynamic systems have also emerged in the field of suicidology showing prominent findings (Schiepek et al., 2011; Fartacek et al., 2016). Non-linear dynamic systems allow to determine short-term predictions by using early warning signs or proximal indicators of suicide (Schiepek et al., 2011). It also allows patient’s monitoring by integrating continuous self-assessment in an internet-based application of suicidality in real time (Fartacek et al., 2016). These results are promising for clinical practice, providing new opportunities for suicide intervention and suicide prevention. Thus, future research should examine these innovative approaches for improving the actual risk assessment and short-term predictions in clinical setting where errors in predictions can result in fatal consequences for patients and mental health system.

Despite these limitations, the currentt study has a number of strengths that increase confidence in the validity of the results including the longitudinal prospective nature of the present study across 18 months of follow-up, the homogeneity of the sample which was restricted to criminal offenders with schizophrenia and other psychosis, the use of multiple dataset from the hospital record system and the inclusion of independent psychologists who were blind to the rating scales and outcomes incidents during the follow-up.

## Conclusion

Suicide and violent behaviors are common adverse outcomes in criminal samples of patients with Schizophrenia and related disorders. Potential interventions based on continuous monitoring, closer supervision, restriction to lethal suicide methods and standardized prevention initiatives should be considered as priority for reducing the risk for violence, suicide and other adverse outcomes in Forensic Psychiatric Hospitals. Given the low predictive value of risk categorizations, particularly for events with low base rates, it is important to anticipate the potential impact of additional interventions by considering all the modifiable risk factors in the individual management plan of each patient (Bouch and Marshall, 2003).

## Notes

Prediction of suicide related events and violent behaviors in a sample of institutionalized offenders with Schizophrenia and other psychosis.

## Author Contributions

MS-S and RF-C conceived and designed the experiments. MS-S and JO-C analyzed the data. MS-S, JB, and MB contributed materials and analysis tools. MS-S and HK wrote the paper. RF-C, JB, JO-C, MB, and HK feedback and revision of the text.

## Conflict of Interest Statement

The authors declare that the research was conducted in the absence of any commercial or financial relationships that could be construed as a potential conflict of interest.
